# The p24 Complex Contributes to Specify Arf1 for COPI Coat Selection

**DOI:** 10.3390/ijms22010423

**Published:** 2021-01-03

**Authors:** Susana Sabido-Bozo, Ana Maria Perez-Linero, Javier Manzano-Lopez, Sofia Rodriguez-Gallardo, Auxiliadora Aguilera-Romero, Alejandro Cortes-Gomez, Sergio Lopez, Ralf Erik Wellinger, Manuel Muñiz

**Affiliations:** 1 Department of Cell Biology, Faculty of Biology, University of Seville, 41012 Seville, Spain; ssabido@us.es (S.S.-B.); anamaripl87@hotmail.com (A.M.P.-L.); jmanzano@us.es (J.M.-L.); srodriguez13@us.es (S.R.-G.); auxi@us.es (A.A.-R.); acgomez@us.es (A.C.-G.); serglom@us.es (S.L.); 2 Instituto de Biomedicina de Sevilla (IBiS), Hospital Universitario Virgen del Rocío/CSIC/Universidad de Sevilla, 41012 Seville, Spain; 3 Centro Andaluz de Biología Molecular y Medicina Regenerativa-CABIMER, Universidad de Sevilla-CSIC-Universidad Pablo de Olavide, 41012 Sevilla, Spain; ralf.wellinger@cabimer.es; 4 Department of Genetics, University of Seville, 41012 Seville, Spain

**Keywords:** p24 complex, COPI, Golgi, ArfGEFs, Arf1

## Abstract

Golgi trafficking depends on the small GTPase Arf1 which, upon activation, drives the assembly of different coats onto budding vesicles. Two related types of guanine nucleotide exchange factors (GEFs) activate Arf1 at different Golgi sites. In yeast, Gea1 in the *cis*-Golgi and Gea2 in the medial-Golgi activate Arf1 to form COPI­coated vesicles for retrograde cargo sorting, whereas Sec7 generates clathrin/adaptor­coated vesicles at the *trans*-Golgi network (TGN) for forward cargo transport. A central question is how the same activated Arf1 protein manages to assemble different coats depending on the donor Golgi compartment. A previous study has postulated that the interaction between Gea1 and COPI would channel Arf1 activation for COPI vesicle budding. Here, we found that the p24 complex, a major COPI vesicle cargo, promotes the binding of Gea1 with COPI by increasing the COPI association to the membrane independently of Arf1 activation. Furthermore, the p24 complex also facilitates the interaction of Arf1 with its COPI effector. Therefore, our study supports a mechanism by which the p24 complex contributes to program Arf1 activation by Gea1 for selective COPI coat assembly at the *cis*-Golgi compartment.

## 1. Introduction

COPI vesicle-mediated retrograde transport along the early secretory pathway is an essential process that recycles resident proteins and anterograde transport factors within the Golgi compartments, and from the Golgi apparatus back to the endoplasmic reticulum (ER). Thereby it contributes to maintain ER homeostasis, to initiate new rounds of ER-to-Golgi vesicle transport and to drive Golgi cisternal maturation [1,2,3,4]. In yeast, COPI vesicles are primarily formed at the earlier Golgi compartments by the small GTPase Arf1 which, upon activation by the functionally redundant exchange factors or ArfGEFs Gea1 and Gea2, is thought to directly recruit and assemble the cytosolic heptameric protein complex COPI for membrane deformation and cargo capture [5,6,7,8,9,10,11,12]. However, the presence of Arf1 is not limited to the early compartments within the Golgi. Indeed, Arf1 also functions at the late Golgi where, upon activation by the ArfGEF Sec7, it recruits other types of coat effectors such as clathrin coat adaptors to form vesicles that export fully processed secretory cargo to the endosomal system, or to the plasma membrane [13,14,15]. The fact that Arf1 is activated in early as well as late Golgi compartments raises the fundamental question of how the active form of Arf1 can preferentially produce COPI vesicles at the early Golgi.

A previous study described an Arf1 activation-independent, direct interaction of Gea1 with the COPI coat [16]. This finding led to postulate a feed-forward mechanism by which the formation of Gea1/COPI coat complexes would channel Arf1 into specifically binding to the COPI effector by triggering Arf1 activation in proximity to the COPI coat. Thus, selective COPI coat assembly by Arf1 at the early Golgi compartments could be then determined by the previous formation or stabilization of Gea1/COPI coat complexes [16,17]. However, since ArfGEF/coat interaction itself has been shown to be insufficient to maintain the coat bound to membranes, additional factors may contribute to specifically stabilize Gea1/COPI coat complexes at the early Golgi membrane [17]. One of these factors could be transmembrane cargos of COPI vesicles such as the p24 proteins, which are abundant and conserved type I transmembrane proteins harboring COPI binding signals on their cytoplasmic tails [18,19,20,21]. They assemble in a heteromeric complex that continuously cycles between the ER and Golgi, being itself a major cargo of COPI retrograde transport vesicles [22,23,24]. Besides acting as a specific cargo receptor during the ER export [25,26], the p24 complex also participates in COPI vesicle formation from the Golgi [5,27,28]. This later role has been mainly attributed to the potential ability of the p24 proteins to stabilize the COPI coat onto the Golgi membrane after Arf1-GTP hydrolysis [5,27,28]. Here, we provide evidence suggesting an even earlier role of the p24 complex in establishing COPI vesicle budding sites. We find that the p24 complex is required to direct Arf1 for specific COPI binding as it promotes the local association of COPI with Gea1. Our results suggest that COPI vesicle cargos, such as the abundant p24 complex, act to spatially delimit the Golgi territory where Arf1 will form COPI retrograde transport vesicles.

## 2. Results

### 2.1. The p24 Complex Specifically Interacts with the ArfGEF Gea1 Through COPI Coat Binding

**Figure 1:**
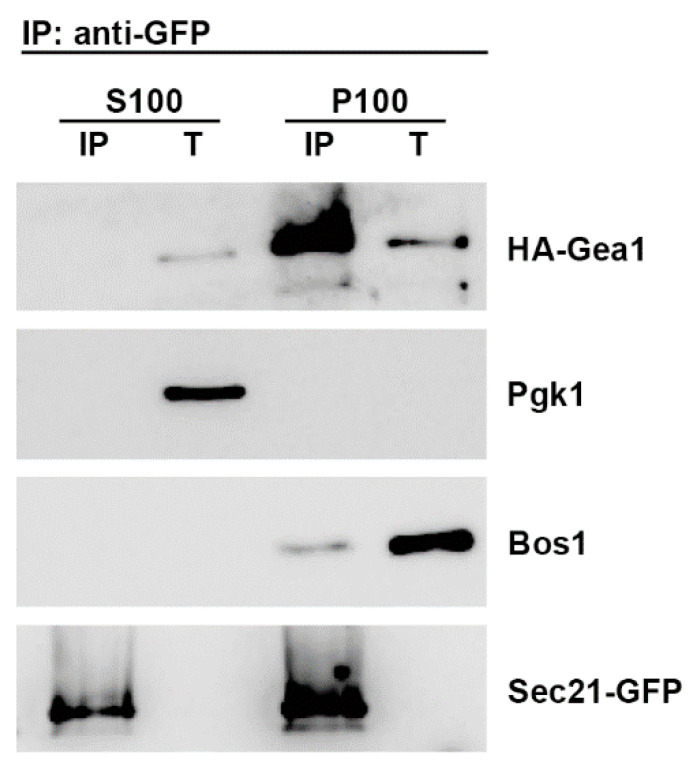
Gea1 and COPI coat specifically interact at the Golgi membrane. Total (T) lysate prepared from wild-type cells expressing HA-Gea1 and the COPI coat subunit Sec21 tagged with GFP was separated into low-speed supernatant (S13) and pellet (P13) fractions by a 13,000× *g* centrifugation step. The S13 enriched-Golgi fraction was further separated into high-speed supernatant (S100) and pellet (P100) fractions by centrifugation at 100,000× *g*. The collected fractions were subjected to specific immunoprecipitation for Sec21-GFP and subsequent immunoblotting using antisera to HA, Pgk1, Bos1 and GFP.

The previous finding, that the COPI coat associates specifically with the ArfGEF Gea1, has led to postulate a feed-forward mechanism by which the COPI coat and Gea1 would form a complex to channel Arf1 activation for COPI vesicle budding [16,17]. We therefore wondered if ArfGEF/coat complexes could be selectively stabilized at the early Golgi by transmembrane COPI vesicle cargos, such as the p24 complex, in order to establish COPI vesicle budding sites. As a first step to evaluate this hypothesis, we tested whether the interaction between Gea1 and COPI coats occurs at the Golgi membrane or in the cytoplasm. Therefore, we examined the subcellular distribution of Gea1/COPI coat complexes by differential fractionation, followed by specific immunoprecipitation of each fraction for the COPI coat subunit Sec21 (yeast γ-COP) tagged with GFP and subsequent immunoblotting for HA-Gea1. As shown in Figure 1, Gea1/COPI coat complexes were mainly found in the membrane fraction, which agrees with the idea that transmembrane proteins might contribute to their formation on the Golgi membrane.

**Figure 2:**
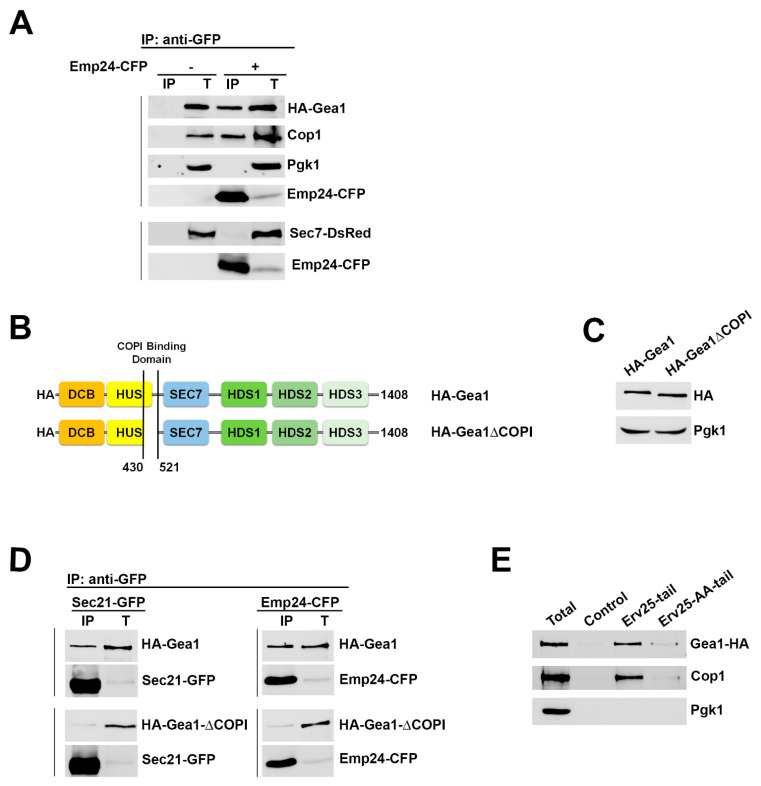
The p24 complex specifically associates with the ArfGEF Gea1 through the COPI coat. (**A**) Emp24-CFP specifically binds the early Golgi-localized ArfGEF HA-Gea1 and the COPI coat subunit Cop1 but not the late Golgi-localized ArfGEF Sec7-dsRed. Native coimmunoprecipitation assay between the p24 complex subunit Emp24-CFP and HA-Gea1, Cop1 or Sec7-DsRed. S13 enriched-Golgi fractions of wild-type cells expressing the indicated tagged proteins were immunoprecipitated with anti-GFP antibody, followed by immunoblotting. Total (T) represents a fraction of the solubilized input material. (**B**) Scheme of full-length HA-Gea1 and deletion construct of HA-Gea1p (HA-Gea1ΔCOPI) lacking residues 434–521, a region involved in direct binding to the COPI coat [16]. DCB, dimerization and cyclophilin binding domain; HUS, homology upstream of Sec7 domain; Sec7, guanine nucleotide exchange factor domain; HDS1, HDS2, HDS3, homology downstream of Sec7 domains. (**C**) HA-Gea1ΔCOPI is expressed to the level of the full-length HA-Gea1. Protein expression levels of each construct were analyzed by Western blot of whole cell extracts. Pkg1 serves as a loading control. (**D**) The lack of the COPI coat binding region (434–521) [16] in HA-Gea1ΔCOPI prevents its efficient association to the p24 complex. S13 enriched-Golgi fractions of wild-type cells expressing indicated tagged proteins were immunoprecipitated with anti-GFP antibody, followed by immunoblotting. Total (T) represents a fraction of the solubilized input material. (**E**) The p24 proteins interact with Gea1 through COPI binding. Synthetic peptides corresponding to the cytoplasmic tail of the p24 protein Erv25 (KNYFKTKHII) or to Erv25-AA with aromatic residues replaced by alanines (KNAAKTKHII) were coupled to thiopropyl-Sepharose beads. These peptide-bound beads and empty beads (Control) were incubated with cytosol from a wild-type strain expressing HA-Gea1. Bound material was resolved by SDS-PAGE and analyzed by immunoblot.

As the p24 proteins efficiently bind the COPI coat [19], we next assessed whether the p24 complex is also able to specifically associate with Gea1. For this purpose, yeast cells expressing HA-Gea1 were analyzed by native coimmunoprecipitation. As seen in Figure 2A, immunoprecipitation in detergent-solubilized extracts revealed that the p24 protein Emp24-CFP coprecipitated with HA-Gea1. As a positive control, binding of Emp24-CFP with the COPI coat subunit Cop1 (yeast α-COP) was also observed. These physical interactions were specific because the unrelated cytosolic protein Pgk1 was not recovered after Emp24-CFP coimmunoprecipitation. If the p24 complex is involved in the selective formation of COPI vesicles from the early Golgi by stabilizing specific complexes containing Gea1 and COPI, it should not interact with Sec7, the other class of ArfGEF that forms clathrin-coated vesicles at the late Golgi [29]. Cells expressing Sec7-dsRed were also analyzed by native coimmunoprecipitation (Figure 2A). As expected, Emp24-CFP was not coprecipitated with Sec7-dsRed, indicating that, specifically, the p24 proteins form a complex only with Gea1 and COPI.

Due to the direct, specific interaction of Gea1 with the COPI coat, we next assessed whether the p24 complex binds Gea1 indirectly through the COPI coat. The Gea1 residues 434–521 are part of the conserved HUS (Homology Upstream Sec7) domain that directly binds the γ subunit of the COPI coat [16]. Thus, we analyzed the interaction of p24 with a HA-Gea1 N-terminal deletion mutant lacking residues 434–521 (HA-Gea1ΔCOPI) by coimmunoprecipitation. (Figure 2B). HA-Gea1ΔCOPI was stably expressed to wild-type levels and showed reduced binding to Sec21-GFP indicating that COPI binds to the 434–521 region of Gea1 as previously shown by Deng et al. (Figure 2C,D) [16]. Furthermore, as seen in Figure 2D, the full-length wild-type HA-Gea1 was efficiently recovered by Emp24-CFP coimmunoprecipitation. However, HA-Gea1ΔCOPI failed to efficiently bind Emp24-CFP. The reduced capacity of HA-Gea1ΔCOPI to bind Sec21-GFP and Emp24-CFP suggests that the p24 complex binds Gea1 through the COPI coat. To confirm this observation, we performed an in vitro binding assay with immobilized p24 tail peptides and cytosol (Figure 2C). Synthetic peptides corresponding to the C-terminal 10 amino acids of the p24 protein Erv25 were coupled to Sepharose beads and incubated with a cytosolic protein extract prepared from wild-type cells expressing HA-Gea1. After the pulldown, both HA-Gea1 and the COPI subunit Cop1 were detected bound to the Erv25 tail sequence. Nevertheless, the binding of these two proteins was significantly reduced when the aromatic residues at −7 and −8 were converted to alanines (YF to AA) in the Erv25 tail sequence. The di-aromatic motif of Erv25 has been shown to be necessary for efficient and direct binding of purified COPI [19]. Therefore, the reduced binding of HA-Gea1 to the mutated Erv25 peptide suggests that Gea1 binds to the p24 cytosolic tails indirectly through COPI.

### 2.2. The Specific Association of the Arf1GEF Gea1 with the COPI Coat is Stabilized by the p24 Complex

**Figure 3:**
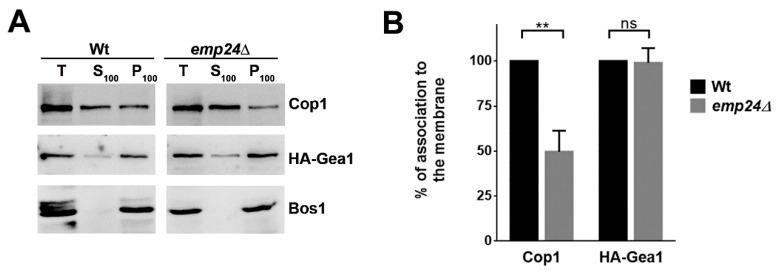
The absence of Emp24 reduces the Golgi membrane association for the COPI coat but not for HA-Gea1. (**A**) Golgi membrane association of Cop1 and HA-Gea1 in wild-type and *emp24Δ* strains. Total (T) lysates prepared from cells expressing HA-Gea1 were differentially centrifuged as in Figure 1. The collected soluble S100 and membrane P100 fractions were analyzed by Western blot using antisera to Cop1, HA and the SNARE Bos1, used as Golgi membrane marker. (**B**) Quantification of three independent experiments. The graph plots the average percentage of the Golgi membrane association of Cop1 and HA-Gea1 normalized to the association in the wild-type strain. Membrane association was determined by quantification of the amount of protein present in the membrane (P100) fraction divided by the amount present in both the membrane and soluble fractions (P100 + S100). Error bars indicate the SD. Statistical significance was determined by using the Student *t* test. ** *p* < 0.01.

Since we found that the p24 complex indirectly interacts with Gea1 through the COPI coat, it was conceivable that the presence of the p24 complex increases the stability of the COPI coat on the early Golgi membrane and thus, promotes the association of Gea1 with COPI coat to initiate COPI vesicle formation. To assess this possibility, we first examined, in vivo, if the yeast p24 complex enhances COPI coat association to the membrane as previously reported for mammalian p24 proteins [30]. For this purpose, we used the deletion of *EMP24* that destabilizes the other proteins of the complex, leading to a complete loss of p24 complex function, and analyzed the subcellular distribution of COPI coat in the absence of the p24 complex by differential fractionation [22]. We thereby observed that Cop1 was less associated to the membrane fraction in the *emp24∆* mutant compared to the wild-type strain (Figure 3). Thus, we next assessed if the p24 complex is also required for the efficient recruitment of HA-Gea1 to the membrane (Figure 3). In contrast to Cop1, the binding of HA-Gea1 to the membrane was not affected by the lack of the p24 complex, suggesting that recruitment of HA-Gea1 is independent of the presence of the COPI coat onto the Golgi membrane.

**Figure 4:**
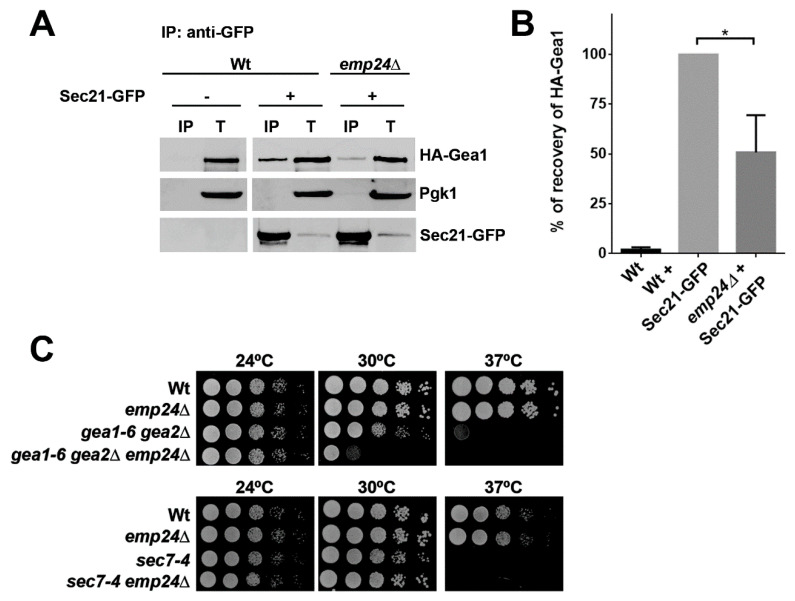
The Gea1/COPI coat complex is stabilized by the p24 complex. (**A**) The p24 complex is required for the efficient interaction between Gea1 and the COPI coat. Detergent-solubilized S13 fractions of wild-type and *emp24Δ* mutant cells expressing HA-Gea1 and the COPI coat subunit Sec21-GFP were subjected to native immunoprecipitation (IP) with anti-GFP antibody, followed by immunoblotting. Total (T) represents a fraction of the solubilized input material. (**B**) Quantification of three independent experiments. The graph plots the average percentage of the recovery of HA-Gea1 normalized to the recovery in the wild-type strain. Error bars indicate the SD. Statistical significance was determined by using the Student *t* test. * *p* < 0.05. (**C**) The loss of the p24 complex intensifies the thermosensitive growth defect in the *gea1-6 gea2Δ* double mutant but not in the *sec7-4* mutant strain. Cells were tested for growth at 24, 30 and 37 °C on YPUAD.

Our finding, that the yeast p24 complex contributes to have more COPI coat associated to the membrane, prompted us to study whether the p24 complex also contributes to the efficient interaction between Gea1 and COPI coat. To address this possibility, we assessed the fraction of HA-Gea1 bound by Sec21-GFP in the absence or presence of the p24 complex. Thereby, we observed significant reduction in the coprecipitation of HA-Gea1 with Sec21-GFP in *emp24∆* mutant extracts as compared to wild-type extracts (Figure 4A,B). These results suggest that the efficient association of Gea1 with the COPI coat depends on the Golgi presence of the p24 complex.

We next strove to see if the p24 complex may participate in the initial stage of COPI vesicle formation by stabilizing the Gea1/COPI coat complex. Under physiological conditions, the existence of many other transmembrane cargos of COPI vesicles can likely attenuate the defect caused by the absence of the p24 proteins. However, when the ArfGEF activity of partially redundant Gea1 and Gea2 proteins is compromised, p24 could become indispensable for COPI vesicle generation. To investigate this possibility, we assessed the viability of *gea1-6 gea2∆* double mutant strains and a *gea1-6 gea2∆ emp24∆* triple mutant strains*2* by drop test analysis (Figure 4C). In line with our idea, the growth of cells carrying the temperature sensitive *gea1-6* allele lacking Gea2 and Emp24 became displayed as a slow growth phenotype and increased temperature sensitivity at 30 °C. As control, we took advantage of a mutant impaired in Sec7 function because Sec7 is the other ArfGEF present at the late Golgi, where it activates Arf1 to produce clathrin-coated vesicles. Interestingly, the *emp24Δ* mutant did not show any synthetic interaction with *sec7-4*, a temperature-sensitive mutant allele of *SEC7*. These results strengthen the idea that the functional interaction between the *EMP24* and *GEA1* is specific for COPI vesicle formation.

### 2.3. The p24 Complex Contributes to Specify Arf1 Activation for COPI Binding

**Figure 5:**
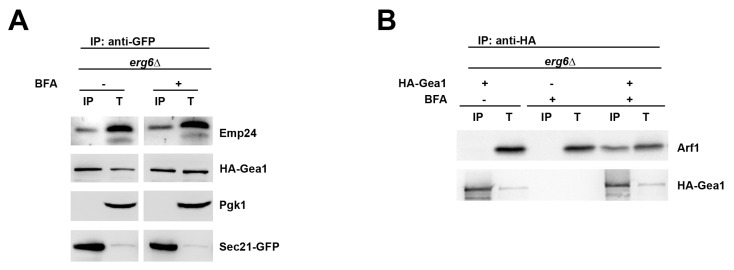
The p24 complex can interact with COPI independently of Arf1 activation. (**A**) *erg6Δ* mutant cells expressing HA-Gea1 and the COPI coat subunit Sec21-GFP were incubated with or without 100 µg/mL BFA (Brefeldin A) for 20 min. *erg6Δ* mutation was used to increase the plasma membrane permeability to BFA. Detergent-solubilized S13 fractions from these cells were subjected to native immunoprecipitation (IP) with anti-GFP antibody, followed by immunoblotting. Total (T) represents a fraction of the solubilized input material. (**B**) BFA treatment is effective because it traps Arf1 in complex with HA-Gea1. *erg6Δ* mutant cells expressing HA-Gea1 were incubated with or without BFA for 20 min. Detergent-solubilized S13 fractions from these cells were subjected to native immunoprecipitation (IP) with anti-HA antibody, followed by immunoblotting. Total (T) represents a fraction of the solubilized input material.

Gea1 was previously shown to interact with the COPI coat subunit Sec21 in the presence of the ArfGEF inhibitor Brefeldin A (BFA) [16], which blocks the GDP/GTP exchange reaction by trapping the Arf1-GDP/GEF complex [31,32,33]. This finding indicates that Gea1/COPI complex formation does not depend on Arf1 activation. Likewise, since the p24 complex facilitates the formation of the Gea1/COPI complex by increasing the COPI association to the membrane, this initial COPI-p24 binding should also occur independently of Arf1 activation. As shown in Figure 5A, coimmunoprecipitation confirms the interaction of Sec21-GFP with Emp24 and HA-Gea1, even in the presence of BFA. Notably, since the BFA treatment was effective because Arf1 was trapped in complex with HA-Gea1 (Figure 5B), this result suggests that COPI can be recruited by the p24 complex to the early Golgi membrane before Arf1 activation.

**Figure 6:**
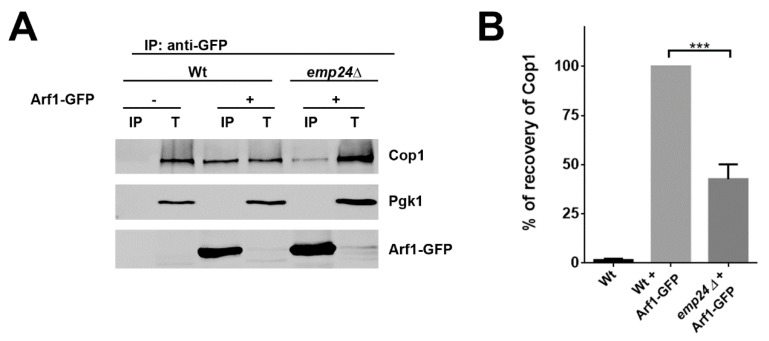
The p24 complex facilitates the interaction of Arf1-GFP with COPI. (**A**) Detergent-solubilized S13 fractions of wild-type and *emp24Δ* mutant cells expressing Arf1-GFP were subjected to native immunoprecipitation (IP) with anti-GFP antibody, followed by immunoblotting. Total (T) represents a fraction of the solubilized input material. (**B**) Quantification of three independent experiments. The graph plots the average percentage of the recovery of Cop1 normalized to the recovery in the wild-type strain. Error bars indicate the SD. Statistical significance was determined by using the Student *t* test. *** *p* < 0.001.

The subsequent association of COPI to Gea1 might then ensure that COPI is already in place when Arf1-GTP is generated by Gea1. Accordingly, the stabilization of the Gea1/COPI complex by the p24 complex should direct Arf1 to bind the proximal COPI effector. We addressed this prediction by determining the amount of Cop1 bound to Arf1-GFP in the absence or the presence of the p24 complex. We observed that in the *emp24∆* mutant, Cop1 was less precipitated by Arf1-GFP than in the wild-type strain (Figure 6). This result further confirms that the p24 complex contributes to specify Arf1 for COPI coat selection at the early Golgi.

## 3. Discussion

**Figure 7:**
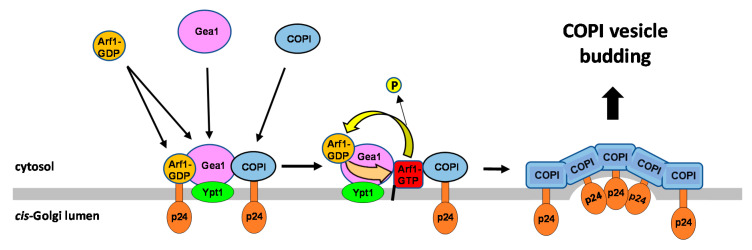
Schematic representation of the proposed role of the p24 complex in programming Arf1 activation by Gea1 for selective COPI coat assembly. COPI can be initially recruited by the p24 complex, a major retrograde cargo of COPI vesicles, to the *cis*-Golgi membrane independently of Arf1 activation. There, the p24 complex and COPI form a transient complex with the ArfGEF Gea1 through direct binding to COPI. Gea1 is separately recruited by the Rab GTPase Ypt1 to the *cis*-Golgi membrane lacking anionic lipids. The p24 complex has been also proposed to facilitate the recruitment of the inactive form of Arf1 (Arf1-GDP). Arf1-GDP could be also recruited by Gea1 as its substrate. The local formation of this transient complex ensures that Arf1 activation by Gea1 occurs in the proximity of COPI, which leads to the COPI coat assembly by the generated Arf1-GTP and the p24 complex. Arf1-GTP can be deactivated by the specific COPI-associated ArfGTPase Glo3 to initiate a new activation cycle by Gea1. Thus, through this feed-forward mechanism, the p24 complex could contribute to establish COPI vesicle budding sites by facilitating the local encounter of COPI, Gea1 and Arf1.

How the same Arf1 GTPase, upon activation, manages to generate different types of coated vesicles, depending on the donor Golgi compartment, remains a central question in Golgi trafficking. Here, we investigated how Arf1 selectively contributes to COPI vesicle formation at the *cis*-Golgi. According to the classical model, different cytosolic coats can be potentially targeted by the activated Arf1-GTP to the *cis*-Golgi membrane but only COPI would be selectively assembled upon additional binding with transmembrane retrograde cargo such as the p24 proteins to form COPI vesicles [5]. Deng et al. suggested an alternative mechanism based on their observation that Gea1, the Arf1-GEF specifically localized at the *cis*-Golgi, directly interacts with the COPI coat in the presence of BFA, thereby, independent of Arf1 activation [16]. This finding led to postulate a feed-forward mechanism by which Gea1/COPI complexes would channel Arf1 activation to select the adjacent COPI coat effector for COPI vesicle budding [17]. Our study supports this model and suggests that the p24 complex, a major cargo of COPI vesicles, contributes to specify Arf1 for COPI selection by stabilizing the Gea1/COPI complexes at the *cis*-Golgi membrane (Figure 7). We show that association of COPI to the *cis*-Golgi membrane is promoted by the p24 complex, while Gea1 is separately recruited by the Rab GTPase Ypt1 to the *cis*-Golgi lacking exposed anionic lipids such as phosphatidylserine (PS) or phosphatidylinositol-4 phosphate (PI4P) [9]. The p24-dependent COPI recruitment to the *cis*-Golgi membrane favors the local encounter of COPI with Gea1, leading to the formation of the transient ternary complex Gea1/COPI/p24. Accordingly, we found that the p24 complex facilitates Gea1/COPI association and indirectly interacts with Gea1 through COPI. Most importantly, we observed that COPI binds to the p24 complex in the presence of the ArfGEF inhibitor BFA. This finding suggests that the COPI coat can be recruited to the *cis*-Golgi membrane via the p24 complex and thereby will interact with Gea1 before Gea1-dependent Arf1 activation. Consistently, COPI was also shown to bind Gea1 in the presence of BFA [16]. The fact that Gea1 can activate Arf1 while simultaneously interacting with COPI should imply that the generated Arf1-GTP binds COPI coat rather than to other coat effectors. Coherent with this prediction, we show that Arf1 binding to COPI is reduced in the absence of the p24 complex. In addition, to facilitate the formation of the Gea1/COPI complex before Arf1 activation, the p24 complex could also play other roles that contribute to the local formation of COPI vesicles at the *cis*-Golgi membrane. First, p24 proteins could initially recruit the deactivated form of Arf1 (Arf1-GDP) [23], facilitating its interaction with the pre-assembled Gea1/COPI complex to be subsequently activated and directed for COPI binding. Furthermore, the direct interaction of p24 proteins with the ArfGAP Glo3 [27], which is stably associated to COPI, could contribute to regulate its functions in COPI vesicle biogenesis and uncoating [34,35,36]. Therefore, we suggest that the ability of p24 proteins to oligomerize could contribute to establish multiple, transient interactions with the different regulatory elements of the COPI vesicle machinery, favoring their local encounter on the *cis*-Golgi membrane for productive vesicle budding (Figure 7).

Our study is consistent with a mechanism that could contribute to the spatial organization of vesicle budding in the maturing Golgi [14,37]. Transmembrane retrograde cargos, such as the abundant p24 proteins, would contribute, through their ability to bind COPI, to the formation of Gea1/COPI complexes on the *cis*-Golgi membrane, which should activate Arf1 for specific COPI binding and thus, to establish COPI vesicle budding sites. An analogous mechanism should operate for Gea2, which has been also shown to bind COPI and is present in later Golgi compartments than Gea1 [16,38]. By using this mechanism, the COPI coat might be able to outcompete with other Arf1 coat effectors for Arf1-GTP binding at the early Golgi compartments where retrograde cargos are mostly present. Therefore, the collective presence of retrograde cargos could contribute to spatially delimit the Golgi territory where Arf1 will form COPI retrograde transport vesicles. In the maturing Golgi, the progressive depletion of retrograde cargo from late Golgi compartments by COPI-dependent recycling could facilitate the Arf1 switch to generate clathrin/adaptor-coated vesicles at the TGN for Golgi export of anterograde secretory cargo. In the absence of retrograde cargo, the recruitment of the Arf1GEF Sec7 to the TGN to activate Arf1 for selective clathrin vesicle budding involves several regulatory mechanisms including the presence of anionic lipids at the membrane surface, autoinhibition, the positive feedback loop between Arf1 and Sec7, the Rab GTPase Ypt1 and the Arf-like GTPase Arl1 [14,39,40]. Interestingly, the anterograde cargo has been also implicated in the establishment of vesicle budding sites at the TGN [41,42]. Therefore, in addition to the extensive crosstalk and feedback between different Arf and Rab GTPases, their GEF and GAP regulatory proteins, effectors and specific membrane lipids [43,44], cargo proteins could also contribute to the directionality of Golgi trafficking. Further work, also including in vitro reconstitution and live cell microscopy techniques, will be necessary to determine the collective role of cargo in the complex regulatory circuitry that controls Golgi maturation.

## 4. Materials and Methods

### 4.1. Media and Growth Conditions

Yeast cultures were grown at 24 °C in rich YP medium (1% yeast extract, 2% peptone) supplemented with 0.2% adenine and containing 2% glucose (YPD) as carbon source or in synthetic minimal medium (0.15% yeast nitrogen base, 0.5% ammonium sulfate) supplemented with the appropriate amino acids and bases as nutritional requirements, and containing 2% glucose (SD) as carbon source.

### 4.2. Yeast Strains and Plasmids

Strains of *Saccharomyces cerevisiae* and plasmids used for this work are listed in Table 1 and Table 2 respectively.

### 4.3. Plasmid Construction

Plasmid pRC4-GEA1-∆COPI contains a *GEA1* allele expressing a truncated Gea1 protein lacking amino acids 434 to 521. To generate plasmid pRC4-GEA1-∆COPI, DNA fragments up and downstream of nucleotides 1302 and 1563 of the *GEA1* ORF were amplified from genomic DNA using overlapping nucleotides. The PCR products were mixed and amplified using external oligonucleotides, digested with XbaI/NheI and inserted into XbaI/NheI site of pRC4, thereby replacing a 1777 kb fragment with a 1516 bp fragment.

### 4.4. Differential Fractionation

Differential fractionation was performed as described by Chen et al. [45] with some modifications. A total of 100 mL of cells was harvested at 5 × 10^6^ cell/mL, washed with 10 mM sodium azide, spheroplasted, lysed, layered on a 1 M sorbitol cushion (1.7 M sorbitol and 50 mM potassium phosphate [pH 7.5]), and centrifuged at 6500× *g* for 3 min. The pellet was lysed in 1 mL of lysis buffer (20 mM HEPES (pH 7.4) with protease inhibitors) and centrifuged at 2500× *g* for 2 min in a microfuge. The supernatant was removed and spun at 13,000× *g* for 30 min. The resultant low-speed supernatant (S13 enriched-Golgi fraction) was further separated into high-speed supernatant (S100) and pellet (P100) fractions by centrifugation at 100,000 × *g*. The S100 supernatant was saved, and the P100 pellet was resuspended in lysis buffer.

### 4.5. Native Coimmunoprecipitation

Native coimmunoprecipitation experiments were performed on enriched-Golgi fractions as follows. 100 OD600 units of yeast cells were washed twice with HEPES buffer (20 mM HEPES (pH 7.2), 100 mM KCl, 5 mM MgCl2, 1 mM phenylmethylsulfonylfluoride, and protease inhibitor cocktail; Roche Diagnostics) and disrupted with glass beads, after which cell debris and glass beads were removed by centrifugation. The supernatant was then centrifuged at 13,000× *g* for 15 min at 4 °C. The resultant supernatant was diluted with HEPES buffer, and Triton X-100 was added to a final concentration of 1%. The suspension was incubated for 60 min at 4 °C with rotation, after which insoluble components were removed by centrifugation at 13,000× *g* for 60 min at 4 °C. For immunoprecipitation of GFP-tagged proteins, the sample was first preincubated with Bab agarose beads (ChromoTek) at 4 °C for 1 h and subsequently incubated with GFP-Trap_A (ChromoTek, Planegg, Germany) at 4 °C for 3 h. The immunoprecipitated beads were washed five times with HEPES buffer containing 0.1% Triton X-100, eluted with SDS sample buffer, resolved on SDS-PAGE, and analyzed by immunoblot.

### 4.6. BFA Treatment

*erg6Δ* mutation was used to increase the plasma membrane permeability to BFA. For BFA treatment, *erg6Δ* cells were incubated at 24 °C in the presence of 100 µg/mL BFA for 20 min. This concentration of BFA was maintained during the subsequent native coimmunoprecipitation [16].

### 4.7. In Vitro Pull-Down Assay

Synthetic peptides corresponding to the 10 C-terminal amino acids of Erv25 (KNYFKTKHII) and Erv25-AA (KNAAKTKHII) with an N-terminal cysteine residue were generated (JPT Peptide Technologies) and linked to thiopropyl-Sepharose 6B (GE Healthcare) as described by Belden and Barlowe [19]. Cytosol from a strain expressing HA-Gea1 was obtained as described by Muñiz et al. [26]. In vitro binding reactions were performed as described by Belden and Barlowe [19].

## Figures and Tables

**Table 1:** *Saccharomyces cerevisiae* strains.

Strain	Genotype	Source
MMY1156	*MATa SEC21-GFP::HIS3 ura3 leu2 his3 lys2 ade2 trp1*	This study
BY4742	*MATα ura3 leu2 his3 lys2*	Euroscarf
Y06829	*MATa gea1Δ::kanMx ura3 leu2 his3 met15*	Euroscarf
MMY835	*MATa gea1Δ::kanMx emp24Δ::HmBx ura3 leu2 his3 met15*	This study
MMY1157	*MATa SEC21-GFP::HIS3 emp24Δ::kanMx ura3 leu2 his3 lys2 trp1*	This study
RSY1818	*MATα ura3 leu2 his3 lys2 ade2*	A. Spang
MMY914	*MATα emp24Δ::kanMx leu2.3,112 ura3-52 his3Δ200*	This study
APY022	*MATα gea2Δ::HIS3 gea1-6 leu2 his3 lys2 ade2 ura3*	A. Spang
MMY816	*MATα gea1-6 gea2Δ::HIS3 emp24Δ::HmBx ura3 leu2 his3 ade2 lys2*	This study
MMY1320	*MATa sec7-4 trp1 ura3 leu2 his3 ade2*	This study
MMY1321	*MATα emp24Δ::kanMx sec7-4 ura3 leu2 his3 ade2*	This study
INV-ARFGFP	*MATa ARF1-GFP::HIS3 ura3Δ0 leu2Δ0 his3Δ1 met15Δ0*	Invitrogen
MMY562	*MATα emp24Δ::KanMx4 ARF1-GFP::HIS3 ura3 leu2 his3*	This study
MMY1668	*MATa erg6∆::hph SEC21-GFP::HIS3*	This study

**Table 2:** *Plasmids*.

Number	Yeast Marker	Yeast Replication	Plasmid	Source
pRC4	URA3	2μ	5xHA-GEA1	C.L. Jackson
pRC4-GEA1-ΔCOPI	URA3	2μ	pRC4-5xHA-GEA1-ΔCOPI	This study
RH3127	URA3	CEN	EMP24-CFP (YCPlac33)	G. Castillón
pRS415	LEU2	CEN	SEC7-dsRED	A. Spang
pYD111	URA3	2μ	YEp352-5xHA GEA1 (5-611)	C.L. Jackson

## Data Availability

The data that support the findings of this study are available from the corresponding author upon reasonable request.

## Abbreviations

GEF	Guanosine Exchange Factor
GAP	GTPase Activating Protein
COPI	Coat Protein Complex I
ARF	ADP-Ribosylation Factor
BFA	Brefeldin A
